# Asian Cohort for Alzheimer’s Disease (ACAD) Study on Genetic and Non‐Genetic Risk Factors for Alzheimer’s Disease among Asian Americans and Canadians

**DOI:** 10.1002/alz.085391

**Published:** 2025-01-09

**Authors:** Li‐San Wang, Pei‐Chuan Ho, Boon Lead Tee, Clara Li, Yian Gu, Jennifer S. Yokoyama, Dolly Reyes‐Dumeyer, Kelley M. Faber, Wan‐Ping Lee, Yeunjoo E. Song, Marian Tzuang, Badri N. Vardarajan, Hyun‐Sik Yang, Yun‐Beom Choi, Howard H. Feldman, Joshua D Grill, Victor W. Henderson, Ging‐Yuek Robin Hsiung, Richard Mayeux, Howard J. Rosen, Rohit Varma, Tatiana M. Foroud, Walter A. Kukull, Guerry M. Peavy, Haeok Lee, W. Haung Yu, Helena C Chui, Gyungah R Jun, Van Ta Park, Tiffany W Chow, The Asian Cohort for Alzheimer’s Disease Study

**Affiliations:** ^1^ University of Pennsylvania Perelman School of Medicine, Philadelphia, PA USA; ^2^ Memory and Aging Center, UCSF Weill Institute for Neurosciences, University of California, San Francisco, San Francisco, CA USA; ^3^ Department of Psychiatry, Icahn School of Medicine at Mount Sinai, New York, NY USA; ^4^ Columbia University Irving Medical Center, New York, NY USA; ^5^ University of California, San Francisco, San Francisco, CA USA; ^6^ Columbia University, New York, NY USA; ^7^ National Centralized Repository for Alzheimer’s Disease and Related Dementias (NCRAD), Indianapolis, IN USA; ^8^ Case Western Reserve University, Cleveland, OH USA; ^9^ University of California San Francisco School of Nursing, San Francisco, CA USA; ^10^ The Taub Institute for Research on Alzheimer’s Disease and The Aging Brain, Columbia University, New York, NY USA; ^11^ Center for Alzheimer’s Research and Treatment, Brigham and Women’s Hospital, Harvard Medical School, Boston, MA USA; ^12^ Englewood Health, Englewood, NJ USA; ^13^ Alzheimer’s Disease Cooperative Study (ADCS), University of California San Diego, La Jolla, CA USA; ^14^ University of California, Irvine, Irvine, CA USA; ^15^ Stanford University School of Medicine, Stanford, CA USA; ^16^ University of British Columbia, Vancouver, BC Canada; ^17^ The Taub Institute for Research on Alzheimer’s Disease and the Aging Brain, Vagelos College of Physicians & Surgeons, Columbia University, New York, NY USA; ^18^ UCSF Weill Institute for Neurosciences, University of California, San Francisco, CA USA; ^19^ Southern California Eye Institute, CHA Hollywood Presbyterian Medical Center, Los Angeles, CA USA; ^20^ Indiana University School of Medicine, Indianapolis, IN USA; ^21^ National Alzheimer’s Coordinating Center, University of Washington, Seattle, WA USA; ^22^ University of California San Diego, Department of Neurosciences, La Jolla, CA USA; ^23^ New York University, New York, NY USA; ^24^ University of Toronto, Toronto, ON Canada; ^25^ Center for Addiction and Mental Health, Toronto, ON Canada; ^26^ Alzheimer’s Disease Research Center, Keck School of Medicine, University of Southern California, Los Angeles, CA USA; ^27^ Boston University School of Public Health, Boston, MA USA; ^28^ Boston University Chobanian & Avedisian School of Medicine, Boston, MA USA; ^29^ Alector, South San Francisco, CA USA

## Abstract

**Background:**

Asian Americans and Asian Canadians (ASACs) are the fastest growing minority group in the US and Canada. However, ASACs are under‐sampled in Alzheimer’s disease (AD) research. To address the need of culturally appropriate clinical protocols and community‐based recruitment approaches for ASACs, the Asian Cohort for Alzheimer’s Disease (ACAD), the first large dementia genetics cohort focusing on Chinese, Korean, and Vietnamese, launched in 2021 to examine genetic and non‐genetic risk factors for AD among ASACs. Our clinical and community‐based participatory research (CPBR) scientists have a long collaborative history and diverse cultural and scientific training backgrounds: both are critical in leading AD and CBPR research.

**Method:**

Upon receipt of an NIA U19 grant in 2023, ACAD has expanded to 9 recruiting sites (7 US and 2 Canadian), a coordinating site, and an analysis site with a centralized data management system. ACAD developed a comprehensive study protocol including community outreach and recruitment strategies, the data collection packet (DCP), pre‐screening and sample collection procedures, and in English, Chinese (Mandarin and Cantonese), Korean, and Vietnamese. To ensure consistency, ACAD implemented a training curriculum for data/sample collect and for culturally appropriate recruitment approaches in collaboration with community partners, clinics, and nursing homes serving Asian communities.

**Result:**

As of December 2023, more than 2,400 people expressed interests in ACAD. A total of 683 of the 899 consented participants completed DCP data into the REDCap (604 Chinese, 54 Korean, and 25 Vietnamese), while 399 saliva samples and 285 blood samples were received. Participants aged 60 –103 years at enrollment, 67% were female, and 47% reported having a college or above education. Currently, ACAD is revising the study protocol in response to feedback received in its pilot phase, including the need to include additional neuropsychological tests and cultural tailored lifestyle questionnaires with an emphasis on immigration experiences.

**Conclusion:**

The ACAD team (including community partners) have learned valuable lessons and demonstrated the feasibility of recruiting ASACs in clinical research. With an expansion plan and in collaboration with other AD research focuses on racial minority populations, insights from ACAD may identify potential novel, population‐specific therapeutic pathways for AD.

